# Influence factors of early childhood caries risk among children aged 1–2 years in Beijing: a prospective cohort study

**DOI:** 10.1038/s41405-026-00432-1

**Published:** 2026-05-02

**Authors:** Shuxi Miao, Mei Zhao, Wen Ren, Wei Chen, Hui Zhang, Min Liu

**Affiliations:** Department of Preventive Dentistry, Beijing Stomatological Hospital, Capital Medical University, Beijing, China

**Keywords:** Dental epidemiology, Preventive dentistry

## Abstract

**Objectives:**

To investigate risk factors for early childhood caries (ECC) among 1–2-year-old children in Beijing and to assess the effect modification of baseline caries experience on the associations between other risk factors and subsequent caries outcomes.

**Methods:**

A 12–24-month prospective study was conducted from 2021 to 2023, a total of 919 participants with valid data were included in this study, with a follow-up rate of 76.9%. Oral health information and related factors were collected through parent-completed questionnaires and clinical dental examinations. Univariate analyses and a zero-inflated negative binomial (ZINB) regression model were used to identify risk factors.

**Results:**

Caries incidence during follow-up was 29.8%, and the mean increase in decayed, missing, and filled primary teeth (Δdmft) was 0.94 ± 1.94. In the full-sample ZINB model, significant predictors of caries risk were frequency of snack intake, frequency of candy consumption, frequency of bedtime tooth brushing, frequency of bottle use at bedtime, and baseline caries status. In the baseline caries-free group, additional significant predictors of incident caries risk were parents’ caries status, frequency of saliva-sharing behavior, frequency of post-meal mouth rinsing, and the presence of additives in daily drinking water. Interaction analyses showed that the effects of parents’ caries status, saliva-sharing frequency, post-meal mouth rinsing frequency, presence of additives in daily drinking water, bedtime brushing frequency, and history of dental attendance on caries risk differed significantly between children with and without baseline caries.

**Conclusion:**

The risk of early childhood caries (ECC) among 1–2-year-old children in Beijing was associated with multiple oral health-related factors. Baseline caries experience was a strong predictor of subsequent caries risk and significantly modified the effects of other risk factors.

## Introduction

Dental caries is one of the most common chronic diseases. It results from the interplay of cariogenic bacteria, dietary carbohydrates, inadequate nutrition, and various social factors across different ages and socioeconomic backgrounds [1]. Early childhood caries (ECC) is defined as the presence of one or more decayed, missing (due to caries), or filled tooth surfaces in any primary tooth in children under 6 years old [2]. ECC can lead to severe pain, tooth loss, low self-esteem, poor academic performance, sleep disturbances, and reduced quality of life [3]. Considering that oral diseases can adversely affect the development of permanent teeth, establishing a healthy oral environment early in life is critical for lifelong oral health. Therefore, preventing ECC is essential for children to maintain oral health, supporting healthy development, and reduce future dental treatment costs [4].

Most research on ECC prevention has focused on preschool children aged 3–5 years, whereas longitudinal evidence on ECC risk factors among infants and toddlers (0–2 years) remains limited. In 2021, the Beijing Stomatological Disease Prevention and Control Institute launched an oral health care program targeting infants and young children. Leveraging Beijing’s well-established primary healthcare network, the program aims to provide cost-effective early ECC screening, dietary and behavioral counseling, and preventive and/or restorative interventions for local children, ultimately reducing the incidence of early childhood caries.

Modern caries management emphasizes personalized prevention based on caries risk assessment (CRA). The AAPD guidance on pediatric caries risk assessment and management indicates that CRA can be used to understand an individual patient’s pathogenic and protective factors, and thereby to tailor the selection and frequency of preventive and/or therapeutic measures [5]. Meanwhile, some studies have suggested that baseline caries experience is a significant determinant of subsequent incident caries. Baseline caries status may reflect the cumulative effects of multiple cariogenic factors, many of which are also associated with future caries risk [6]. Consequently, the same oral health behavior may exert effects of differing magnitude among children with different “baseline caries status”. If only the average main effects of behaviors are estimated, without evaluating effect modification, important heterogeneity may be obscured, potentially limiting the ability of risk assessment and intervention strategies to achieve genuine stratification and precision prevention.

This study was designed to address two questions: (1) the determinants of ECC risk among infants and toddlers in Beijing, including hereditary factors as well as dietary and oral hygiene behaviors; and (2) whether baseline caries experience exerts a modifying effect on the associations between other factors and ECC risk. The overarching aim was to enable the program to identify children who are susceptible to caries and to support risk-stratified management and more efficient allocation of healthcare resources.

## Materials and methods

### Study population

This study was conducted between 2021 and 2023 and was nested within two routinely implemented community-based oral public health programs. The baseline survey was embedded in the “Beijing Comprehensive Health Care Programme for Children Aged 0–3 Years”. Clinical examinations were conducted in a clinical setting, and questionnaire data were collected from parents through a WeChat mini-program. The program was designed for children aged 0–3 years and their parents in communities across Beijing. Inclusion criteria were: (1) the child had at least one healthy primary tooth; (2) written informed consent provided by the parent; and (3) the child was generally healthy and able to cooperate with oral examination. Exclusion criteria were: (1) the child had no healthy teeth; (2) the child was uncooperative during examination; (3) the caregiver was unable to complete the questionnaire; or (4) the child had systemic disease. The follow-up examination was embedded in the “Beijing Fluoride Foam Caries Prevention Programme” and was conducted 12–24 months after baseline, using the same inclusion and exclusion criteria and under the same examination conditions as the baseline examination.

### Questionnaire survey

The parent questionnaire covered the child’s general information, parents’ caries status, feeding practices, dietary habits, oral hygiene behaviors, and history of dental attendance. (The questionnaire items are detailed in Table 1).

**Table 1:** Oral examination results in the study children (baseline and follow-up).

	Baseline	Follow-up	Incident
Age (month)	26.66 ± 5.00	44.91 ± 4.71	17.78 ± 3.67
Prevalence of dental caries, *n* (%)	283 (30.8)	427 (46.5)	
dmft score (*M* ± *SD*)	1.29 ± 2.43	2.24 ± 3.39	
Caries incidence, *n* (%)			274 (29.8)
∆dmft (*M* ± *SD*)			0.94 ± 1.94

### Oral examination

Oral examinations were conducted in community dental clinics under the supervision of the Beijing Stomatological Disease Prevention and Control Institute. Children’s caries status was measured using the dmft index (decayed, missing due to caries, and filled primary teeth) according to WHO criteria. A tooth was recorded as decayed (dt) if it had a visible cavity, undermined enamel, or detectably softened wall or floor. Teeth extracted due to caries were recorded as missing (mt), and teeth permanently filled with no decay were recorded as filled (ft). Each child’s baseline DMFT score ranged from 0 to 20.

### Indicator definition

Incident caries was defined as the appearance of decay on a previously unaffected primary tooth. Δdmft was defined as the difference between the dmft score at follow-up and at baseline. Caries incidence was defined as the percentage of children with Δdmft > 0.

### Quality control

Oral examinations were conducted in multiple community primary healthcare facilities by local clinicians. To standardize caries assessment across examiners, we developed a standardized examination protocol and diagnostic criteria in accordance with the World Health Organization (WHO) standards from the Oral Health Surveys: Basic Methods (5th edition). Prior to data collection, clinicians received centralized training (online/offline) and were provided with a manual specifying examination procedures, instruments and lighting conditions. During fieldwork, regular supervision and query-resolution procedures were used to address ambiguous cases.

### Data analysis

Data analysis was performed using SPSS 30.0 (IBM, Armonk, NY, USA), Stata 18.0 (StataCorp, College Station, TX, USA), and Python 3.12.7 with the statsmodels and scikit-learn libraries. Pearson’s chi-square test was used to evaluate differences in caries incidence between categorical groups. Because the caries increment (Δdmft) was not normally distributed, a non-parametric Mann–Whitney U test was used to compare the distribution of Δdmft between groups. We conducted Vuong and likelihood-ratio tests to select the best-fitting model among Poisson, negative binomial, zero-inflated Poisson, and zero-inflated negative binomial models. The zero-inflated negative binomial (ZINB) model was chosen as optimal for our data. Variance inflation factors (VIF) were calculated under a linear regression framework to check multicollinearity. We included all candidate predictor variables in both the negative binomial component and the zero-inflation component of the ZINB model. Stepwise backward elimination based on the Akaike Information Criterion (AIC) was applied to simplify the model, stopping when the minimum AIC was achieved. To assess whether the associations between risk factors and subsequent caries risk varied by baseline caries status, a separate model was fitted for children who were caries-free at baseline (Model 2), and, in the full sample, the effects of these risk factors were compared between children who were caries-free at baseline and those with caries at baseline (Model 3).

#### Ethical approval and consent

The study protocol was approved by the Ethics Committee of Beijing Stomatological Hospital, Capital Medical University (Project No. CSA-ICP2022-07). The protocol included the research plan and informed consent forms. Before enrollment, all parents signed informed consent forms, and they received written feedback on the oral examination results for their child.

## Results

### Study participants, ECC characteristics, and univariate analyses

At baseline, a total of 1195 eligible children were enrolled. The follow-up rate was 76.9%, and the 276 children lost to follow-up were mainly lost due to school transfer or absenteeism. No significant differences were observed between children lost to follow-up and those who completed follow-up with respect to questionnaire data or baseline oral examination findings (*χ*² test, *P* > 0.05). A total of 919 children were included in the analysis, of whom 480 (52.2%) were boys and 439 (47.8%) were girls. At the baseline examination, the children’s mean (SD) age was 2.3 (0.4) years. The caries prevalence at baseline was 30.8%, and the mean (SD) dmft score was 1.29 (2.43). At the follow-up examination, the mean (SD) age was 3.8 (0.4) years, and the caries prevalence had increased to 46.5%, with a mean (SD) dmft of 2.24 (3.39). The mean (SD) interval between the two exams was 1.5 (0.3) years. Over the study period, caries incidence was 29.8% and the mean (SD) Δdmft was 0.94 (1.94). The distribution of Δdmft was highly skewed (skewness = 2.68) (Table 1).

Univariate analyses (Table 2) showed that children who frequently fell asleep with bottle, less frequently rinsed their mouth after eating, consumed snacks ≥2 times/day, consumed candy ≥1 time/day, brushed less often before bedtime, or had caries at baseline all had significantly higher caries incidence (*χ*² test, *P* < 0.001) and greater Δdmft (Mann–Whitney *U* test *p* < 0.001).

**Table 2:** Children distribution according to the caries incidence (*n* = 919).

Variables	Number of children, *n* (%)	Caries incidence (%)	*P*	∆dmft (*M* ± *SD*)	*P*
**Sex**			0.242^a^		0.227^b^
male	480 (52.2)	28.1		0.87 ± 1.83	
female	439 (47.8)	31.7		1.02 ± 2.05	
**Parents’ caries status**			0.074^a^		0.103^b^
At least one parent had caries experience	577 (62.8)	31.9		0.98 ± 1.91	
Both parents were caries-free	342 (37.2)	26.3		0.88 ± 2.00	
**Saliva-sharing behavior**			0.132^a^		0.123^b^
≥sometimes	580 (63.1)	31.6		1.02 ± 2.04	
almost never	339 (36.9)	26.8		0.81 ± 1.75	
**Bottle use at bedtime**			<0.001^a^		<0.001^b^
Frequent	81 (8.8)	51.9		1.88 ± 2.77	
Discontinued/rare	838 (91.2)	27.7		0.85 ± 1.82	
**Post-meal mouth rinsing**			<0.001^a^		<0.001^b^
Infrequent/rare	479 (52.1)	35.3		1.13 ± 2.05	
Frequent	440 (47.9)	23.9		0.74 ± 1.79	
**Additives in daily drinking water**			0.224^a^		0.145^b^
Warm boiled water + other additives	128 (13.9)	34.4		1.23 ± 2.29	
Warm boiled water only	791 (86.1)	29.1		0.90 ± 1.88	
**Snacks intake**			<0.001^a^		<0.001^b^
≥2 times/day	299 (32.5)	40.8		1.45 ± 2.44	
≤1 time/day	620 (67.5)	24.5		0.70 ± 1.60	
**Candy consumption**			<0.001^a^		<0.001^b^
≥1 time/day	145 (10.2)	49.7		1.50 ± 1.99	
<1 time/day	774 (84.2)	26.1		0.84 ± 1.91	
**Bedtime toothbrushing**			<0.001^a^		<0.001^b^
Infrequent/rare	224 (24.4)	42.4		1.55 ± 2.46	
Frequent	695 (75.6)	25.8		0.75 ± 1.70	
**Toothpaste use**			0.196^a^		0.179^b^
No	216 (23.5)	33.3		1.05 ± 1.94	
Yes	703 (79.5)	28.7		0.91 ± 1.94	
**Dental floss use**			0.639^a^		0.472^b^
Irregular use/never used	867 (94.3)	30.0		0.96 ± 1.95	
Regular use	52 (5.7)	26.9		0.71 ± 1.73	
**History of dental attendance**			0.388^a^		0.399^b^
Yes	263 (28.6)	27.8		0.87 ± 1.88	
No	656 (71.4)	30.6		0.97 ± 1.96	
**Baseline caries status**			<0.001^a^		<0.001^b^
Caries present	283 (30.8)	45.9		1.53 ± 2.32	
Caries-free	636 (69.2)	22.6		0.68 ± 1.68	

^a^ χ² test.

^b^ Mann–Whitney U test.

### zero-inflated negative binomial (ZINB) regression of ECC risk factors

Vuong’s tests indicated that a zero-inflated Poisson model fit significantly better than a standard Poisson model (*z* = 9.89, *P* < 0.001), and a zero-inflated negative binomial (ZINB) fit better than a standard negative binomial (*z* = 5.18, *P* < 0.001). A likelihood ratio test further showed that the ZINB model (with dispersion parameter *α* ≈ 0.45) provided a superior fit over the zero-inflated Poisson model (*P* < 0.001). Variance inflation factors (VIF) were calculated under a linear regression framework to check multicollinearity; all predictors had VIF < 1.18 (mean 1.08), indicating no significant multicollinearity.

After stepwise reduction, the final ZINB model for the full sample (Table 3, Model 1) achieved an AIC of 2101.92 which reduced from 2123.72. The results from the negative binomial part indicated that snack intake frequency ≥2/day (IRR = 1.43; 95% CI 1.13–1.82; *p* < 0.01) and less frequent bedtime brushing (IRR = 1.37; 95% CI 1.08–1.74; *p* < 0.05) significantly predicted larger Δdmft. In the zero-inflation component, frequent bottle use at bedtime (OR = 0.42; 95% CI 0.21–0.83; *p* < 0.05), snack frequency ≥2/day (OR = 0.65; 95% CI 0.44–0.96; *p* < 0.05), candy frequency ≥1/day (OR = 0.38; 95% CI 0.22–0.66; *p* < 0.001), less frequent bedtime brushing (OR = 0.61; 95% CI 0.40–0.92; *p* < 0.05), and having caries at baseline examination (OR = 0.38; 95% CI 0.26–0.57; *p* < 0.001) were all associated with a higher probability of being in the “susceptible to new caries” group.

**Table 3:** Model 1: Risk factors for ECC in all surveyed children (ZINB regression model, *n* = 919).

negative binomial	Variables	Incidence rate ratio (IRR)	95%CI	*P*
	**Snacks intake**			
	≥2 times/day	1.43	1.13–1.82	0.003
	≤1 time/day (reference)			
	**Candy consumption**			
	≥1 time/day	0.90	0.69–1.17	0.416
	<1 time/day (reference)			
	**Bedtime toothbrushing**			
	Infrequent/rare	1.37	1.08–1.74	0.010
	Frequent (reference)			
	**Dental floss use**			
	Irregular use/never used	1.41	0.79–2.49	0.241
	Regular use (reference)			
	**const**	1.42	0.77–2.62	0.264
**zero-inflated**	**Variables**	**Odds ratio (OR)**	**95%CI**	***P***
	**Bottle use at bedtime**			
	Frequent	0.42	0.21–0.83	0.012*
	Discontinued/rare (reference)			
	**Post-meal mouth rinsing**			
	Infrequent/rare	0.72	0.50–1.03	0.069
	Frequent (reference)			
	**Snacks intake**			
	≥2 times/day	0.65	0.44–0.96	0.028
	≤1 time/day (reference)			
	**Candy consumption**			
	≥1 time/day	0.38	0.22–0.66	0.001
	<1 time/day (reference)			
	**Bedtime toothbrushing**			
	Infrequent/rare	0.61	0.40–0.92	0.019
	Frequent (reference)			
	**Dental floss use**			
	Irregular use/never used	1.21	0.49–2.98	0.672
	Regular use (reference)			
	**Baseline caries status**			
	Caries present	0.38	0.26–0.57	0.000
	Caries-free (reference)			
	**const**	1.48	0.55–3.96	0.439

For the subset of children who were caries-free at baseline (Table 4, Model 2), a separate ZINB model was fitted. After model reduction, the final Model 2 had an AIC of 1190.52, which reduced from 1201.64. The results from the negative binomial part indicated that significant predictors of Δdmft were: at least one parent with caries (IRR = 1.53; 95% CI 1.12–2.09; *p* < 0.01), frequent saliva-sharing behavior (IRR = 1.39; 95% CI 1.03–1.89; *p* < 0.05), frequent bottle use at bedtime (IRR = 1.59; 95% CI 1.08–2.35; *p* < 0.05), less frequent post-meal mouth rinsing (IRR = 1.51; 95% CI 1.10–2.08; *p* < 0.05), presence of additives in daily drinking water (IRR = 1.97; 95% CI 1.35–2.89; *p* < 0.001), and less frequent bedtime brushing (IRR = 1.60; 95% CI 1.18–2.18; *p* < 0.05). In the zero-inflated part, children whose snack intake frequency ≥2/day had a higher probability of being susceptible to new caries (OR = 0.56; 95% CI 0.35–0.90; *p* < 0.05).

**Table 4:** Model 2: Risk factors for ECC of baseline caries-free children (ZINB regression model, *n* = 636).

negative binomial	Variables	Incidence rate ratio (IRR)	95%CI	*P*
	**Parents’ caries status**			
	At least one parent had caries experience	1.53	1.12–2.09	0.008
	Both parents were caries-free (reference)			
	**Saliva-sharing behavior**			
	≥sometimes	1.39	1.03–1.89	0.034
	almost never (reference)			
	**Bottle use at bedtime**			
	Frequent	1.59	1.08–2.35	0.019
	Discontinued/rare (reference)			
	**Post-meal mouth rinsing**			
	Infrequent/rare	1.51	1.10–2.08	0.011
	Frequent (reference)			
	**Additives in daily drinking water**			
	Warm boiled water + other additives	1.97	1.35–2.89	<0.001
	Warm boiled water only (reference)			
	**Snacks intake**			
	≥2 times/day	1.13	0.82–1.56	0.453
	≤1 time/day (reference)			
	**Candy consumption**			
	≥1 time/day	0.97	0.67–1.40	0.874
	<1 time/day (reference)			
	**Bedtime toothbrushing**			
	Infrequent/rare	1.60	1.18–2.18	0.002
	Frequent (reference)			
	**Dental floss use**			
	Irregular use/never used	1.05	0.58–1.88	0.881
	Regular use (reference)			
	**const**	0.74	0.36–1.53	0.420
**zero-inflated**	**Variables**	**Odds ratio (OR)**	**95%CI**	**P**
	**Bottle use at bedtime**			
	Frequent	0.52	0.25–1.09	0.083
	Discontinued/rare (reference)			
	**Snacks intake**			
	≥2 times/day	0.56	0.35–0.90	0.016
	≤1 time/day (reference)			
	Candy consumption			
	≥1 time/day	0.53	0.28–1.01	0.054
	<1 time/day (reference)			
	**Bedtime toothbrushing**			
	Infrequent/rare	0.77	0.47–1.26	0.294
	Frequent (reference)			
	**Dental floss use**			
	Irregular use/never used	1.68	0.64–4.43	0.296
	Regular use (reference)			
	**History of dental attendance**			
	Yes	1.60	0.96–2.69	0.074
	No (reference)			
	**const**	1.95	0.71–5.37	0.196

Interaction analyses (Table 5) showed that for the negative binomial component, the effects of parents’ caries status (*β* = –0.89; *p* < 0.001), saliva-sharing frequency (*β* = –0.62; *p* < 0.01), post-meal rinsing frequency (*β* = –0.74; *p* < 0.01), presence of additives in daily drinking water (*β* = –0.61; *p* < 0.05), and bedtime brushing frequency (*β* = –0.51; *p* < 0.05) on Δdmft were significantly greater in children without baseline caries than in those with baseline caries. In the zero-inflation component, having a history of dental attendance had a significantly stronger positive effect on the probability of new caries among children without baseline caries (*β* = –1.09; *p* < 0.05). Complete results of the interaction model (Model 3) are provided in Table 6.

**Table 5:** Interaction effects of baseline caries status with other variables.

Variables	Negative binomial		Zero-inflated	
	*β*	*P*	*β*	*P*
**Parents’ caries status**	−0.89	0.000*	−0.32	0.456
**Saliva-sharing behavior**	−0.62	0.010*	0.13	0.777
**Bottle use at bedtime**	−0.60	0.052	−0.31	0.687
**Post-meal mouth rinsing**	−0.74	0.003*	−0.40	0.350
**Additives in daily drinking water**	−0.61	0.037*	−0.28	0.639
**Snacks intake**	0.17	0.481	0.08	0.850
**Candy consumption**	−0.37	0.142	−0.88	0.151
**Bedtime toothbrushing**	−0.51	0.031*	−0.89	0.083
**Toothpaste use**	0.03	0.898	−0.01	0.990
**Dental floss use**	0.67	0.303	−1.15	0.221
**History of dental attendance**	−0.27	0.318	−1.09	0.025*

^*^P < 0.05(WALD test).

**Table 6:** Model3 ZINB model in the full sample incorporating interaction terms between baseline caries status and other variables (*n* = 919).

negative binomial	Variables (High risk vs reference)	Baseline caries status	Incidence rate ratio (IRR)	95%CI	*P*	*P* for interaction
	**Baseline caries status**	Caries-free				
		Caries present	4.08	1.00−16.58	0.049	
	**Parents’ caries status**	Caries-free	1.53	1.09–2.14	0.014	<0.001
		Caries present	0.63	0.45–0.87	0.005	
	**Saliva-sharing behavior**	Caries-free	1.37	0.97–1.93	0.073	0.010
		Caries present	0.74	0.53–1.02	0.062	
	**Bottle use at bedtime**	Caries-free	1.68	1.12–2.52	0.012	0.052
		Caries present	0.92	0.58–1.45	0.715	
	**Post-meal mouth rinsing**	Caries-free	1.47	1.03–2.09	0.034	0.003
		Caries present	0.70	0.51–0.97	0.032	
	**Additives in daily drinking water**	Caries-free	1.95	1.30–2.91	0.001	0.037
		Caries present	1.06	0.71–1.59	0.771	
	**Snacks intake**	Caries-free	1.10	0.79–1.54	0.570	0.481
		Caries present	1.30	0.95–1.78	0.103	
	**Candy consumption**	Caries-free	1.00	0.69–1.46	0.998	0.142
		Caries present	0.69	0.50–0.95	0.024	
	**Bedtime toothbrushing**	Caries-free	1.75	1.25–2.45	0.001	0.031
		Caries present	1.05	0.76–1.44	0.784	
	**Toothpaste use**	Caries-free	0.87	0.61–1.24	0.429	0.898
		Caries present	0.90	0.62–1.29	0.555	
	**Dental floss use**	Caries-free	1.11	0.60–2.04	0.740	0.303
		Caries present	2.17	0.70–6.70	0.177	
	**History of dental attendance**	Caries-free	1.21	0.81–1.81	0.354	0.318
		Caries present	0.92	0.65–1.30	0.651	
**zero-inflated**	**Variables (High risk vs reference)**		**Odds ratio (OR)**	**95%CI**	***P***	**P for interaction**
	**Baseline caries status**	Caries-free				
		Caries present	3.83	0.48–30.42	0.203	
	**Parents’ caries status**	Caries-free	0.96	0.60–1.56	0.884	0.456
		Caries present	0.70	0.35–1.39	0.310	
	**Saliva-sharing behavior**	Caries-free	1.12	0.69–1.81	0.659	0.777
		Caries present	1.26	0.61–2.61	0.527	
	**Bottle use at bedtime**	Caries-free	0.53	0.25–1.11	0.094	0.687
		Caries present	0.39	0.11–1.43	0.155	
	**Post-meal mouth rinsing**	Caries-free	0.88	0.54–1.41	0.589	0.350
		Caries present	0.59	0.29–1.17	0.132	
	**Additives in daily drinking water**	Caries-free	1.19	0.66–2.17	0.565	0.639
		Caries present	0.90	0.34–2.42	0.842	
	**Snacks intake**	Caries-free	0.55	0.34–0.89	0.015	0.850
		Caries present	0.60	0.29–1.22	0.158	
	**Candy consumption**	Caries-free	0.52	0.27–1.01	0.053	0.151
		Caries present	0.22	0.08–0.60	0.003	
	**Bedtime toothbrushing**	Caries-free	0.86	0.51–1.46	0.579	0.083
		Caries present	0.35	0.15–0.83	0.016	
	**Toothpaste use**	Caries-free	0.79	0.46–1.35	0.389	0.990
		Caries present	0.78	0.32–1.91	0.594	
	**Dental floss use**	Caries-free	1.72	0.64–4.61	0.278	0.221
		Caries present	0.54	0.11–2.60	0.445	
	**History of dental attendance**	Caries-free	1.73	1.00–2.99	0.050	0.025
		Caries present	0.58	0.26–1.27	0.173	

## Discussion

In this study of children aged 1–2 years in Beijing, we identified several risk factors significantly associated with the development of early childhood caries (ECC) and demonstrated a significant modifying effect of baseline caries status on the impact of these risk factors. Using a zero-inflated negative binomial (ZINB) regression model, we found that certain behavioral factors (e.g., frequent snack consumption and inadequate oral cleaning) were associated with a significantly higher risk of incident ECC. Notably, however, the strength of these associations depended on the child’s baseline caries status. Among children who were caries-free at baseline, adverse dietary habits and suboptimal oral hygiene behaviors were closely associated with new caries onset during follow-up, whereas among children with caries at baseline, the associations between these behavioral factors and further increments in caries were markedly attenuated or non-significant. These findings highlight baseline caries experience as an effect modifier, reshaping the observed relationships between specific oral health behaviors and subsequent caries progression.

The relationship between children’s oral health behaviors and caries has been well-studied, but most research has focused on preschool-aged children (3–5 years old) and is often cross-sectional in design [7]. Longitudinal studies on oral hygiene behaviors and caries risk in infants and young toddlers are relatively few. Tashiro et al. [8], Syafri̇za et al. [9], and Wigen et al. [10] conducted oral examinations and questionnaire surveys among infants and toddlers aged 0–2 years and investigated the associations between the above factors and new developed caries during follow-up. These and most similar studies primarily relied on univariate comparisons or multivariable logistic regression for analysis, which may have limited statistical power. In the present study, we initially attempted to use a multivariable logistic regression to predict caries incidence, but due to the highly unbalanced outcome (many children had no incident caries), the logistic model’s sensitivity was effectively 0. Given the large number of zero values in dmft and the over-dispersion of incident dmft score, Böhning et al. [11] and Lewsey et al. [12] introduced the zero-inflated negative binomial model to dental caries research. This model assumes the population consists of “not susceptible to new caries” children and “susceptible” children and models each subpopulation separately. Compared to traditional linear models or standard Poisson regression, the ZINB model has been shown to provide a better fit for caries count data [13]. To our knowledge, the present study is the first to apply a ZINB model in a longitudinal study of caries risk among infants and young toddlers.

Our findings confirm that baseline caries experience is a strong predictor of future caries risk, consistent with previous studies [14]. In Model 1, children who were caries-free at baseline had 0.38 times the odds of being classified as caries-susceptible compared with those with caries at baseline (OR = 0.38). In Model 3, when all behavioral variables were at their reference levels, children with caries at baseline exhibited approximately a four-fold greater caries increment during follow-up than those who were caries-free at baseline (IRR = 4.08). Our results further indicate that baseline caries experience is not merely an independent risk factor; rather, it represents an integrated reflection of tooth susceptibility to caries, the plaque micro-ecosystem, diet, hygiene practices, fluoride use, dental treatment, and other caries-related factors [15]. Accordingly, baseline caries experience may, to some extent, “reshape” the relative contribution of other behavioral factors to incident caries risk. Among children who were caries-free at baseline, parental caries history, rinsing after meals, and daily drinking-water habits were significantly associated with caries increment. However, these associations were attenuated among children with caries at baseline and, in some instances, even appeared as a reversed “protective” effect. Saliva-sharing behaviors—an established pathway for the vertical transmission of mutans streptococci—showed significant differences in their association with caries increment between children with different baseline caries status [16]. This is consistent with the findings of O’Sullivan and colleagues, who reported that high baseline mutans streptococci levels more strongly predicted caries increment among children who were initially caries-free [17]. Collectively, these findings provide statistical support for developing stratified and graded prevention strategies: for children who are caries-free at baseline, behavioral interventions and family-based oral hygiene management are critical for reducing future caries risk.

The frequency of bedtime brushing, the frequency of snacking, and the frequency of falling asleep with a bottle were all significant risk factors in both Model 1 and Model 2. Snacks commonly introduce sticky carbohydrates [18, 19]; nocturnal bottle use results in prolonged carbohydrate exposure during periods of low oral activity [20, 21]; and infrequent bedtime toothbrushing promotes dental plaque accumulation [22, 23]. The increased caries risk attributable to these factors has been confirmed in multiple studies. Notably, bedtime toothbrushing showed a significant association with the caries incidence among children with caries at baseline, but not with their caries increment; in contrast, the pattern was reversed among children who were caries-free at baseline. This suggests that once the disease process has been initiated, behavioral modification alone may be insufficient to halt further progression. In such cases, clinical preventive measures should be incorporated, such as fissure sealants and professionally applied fluoride [5].

In Model 3, we observed that some poor oral habits were associated with negative coefficients for the dmft increment. This does not mean that these behaviors are protective against caries; rather, it likely reflects the combined effects of index event bias and a ceiling effect. On one hand, both baseline caries status and caries increment are influenced by numerous risk factors. This interrelation can introduce index event bias, causing the contribution of a given behavior to the caries increment to be significantly underestimated or even appear reversed [24, 25]. On the other hand, children with caries at baseline may have already accumulated a high dmft score, leaving limited room for new lesions—a ceiling effect [26]. As a result, their dmft increment during the follow-up is small, and in the model this manifests as a “spurious protective association” for that behavior. Understanding these biases is important when interpreting the model results, to avoid misclassification of harmful behaviors as seemingly protective in certain subgroups.

We did not observe a significant protective effect of floss use or toothpaste use, which contrasts with some previous studies [27]. This discrepancy may be partly explained by limitations inherent in the questionnaire survey. To maintain broad program coverage and practical feasibility, the questionnaire could not be overly complex. As a result, information on the fluoride concentration of the toothpaste, frequency of use, and the effectiveness of toothbrushing was not further verified. In addition, interproximal contacts in the primary dentition typically become established more tightly at around 3–4 years of age; therefore, in this younger cohort, the benefits of flossing may not be statistically detectable. With respect to dental attendance, a history of dental visits showed a protective tendency in the caries-free group but was associated with higher risk in the baseline caries-present group. This pattern may reflect indication bias, whereby dental visits among caries-free children are typically preventive, whereas those among children with existing caries are more often treatment-driven [28]. Future risk assessment should distinguish preventive attendance from problem-oriented visits.

This study was nested within two large-scale community-based oral public health programs routinely implemented in Beijing, whose primary objective was to reduce the prevalence of dental caries among children through sustainable public health services. Owing to the high population mobility in the study area, the rate of loss to follow-up was relatively high. In addition, the study sample was not derived from a standard probability sampling procedure; therefore, the representativeness of the sample with respect to the target population remains uncertain. At present, some longitudinal studies of dental caries have been conducted on the basis of large-scale registry data, electronic health records, or routine public oral health service databases, with methodological emphasis typically placed on data source, outcome definition, follow-up pathway, and statistical control rather than examiner calibration specifically implemented for research purposes [29, 30]. Similarly, although formal examiner calibration was not available in the present study, the data were generated through a large-scale public health service process and therefore still retain research value. In the context of the long-term implementation of public health programs, this exploratory work could in the future be extended to larger samples and longer observation periods to further enhance the robustness of the findings.

A further methodological consideration in the present study was the choice of caries diagnostic criteria. Compared with the WHO criteria, ICDAS is more sensitive in detecting early carious lesions; however, it is also more complex and requires more extensive training and examiner calibration to ensure reliable and consistent assessments. In large-scale primary care or community-based oral screening, the WHO criteria are more feasible to implement and more likely to yield stable and consistent examination results, although they may underestimate early lesions. Despite this limitation, significant associations were still identified between the selected caries risk factors and caries outcomes, indicating that the main findings of this study were not substantively affected by the diagnostic criteria adopted.

Compared with other caries risk studies, we collected relatively rich questionnaire data; however, as the information relied on parental self-report, it may be subject to recall bias and social desirability bias. In terms of social background characteristics, the questionnaire in the present study recorded only parental caries status. Some studies have suggested that maternal caries status may represent a more direct family-level marker of a child’s caries risk, as it may capture the combined influence of maternal oral health behaviors, knowledge, and other social determinants [31, 32]. However, a considerable body of literature has also demonstrated significant associations of ECC risk with factors such as maternal educational level, household income, and parental smoking. Therefore, the assessment of sociodemographic and parental factors in this study remains incomplete and should be expanded in future research. Furthermore, in order to reduce demands on human resources, financial resources, and time while maximizing program coverage, no biological assessments were performed and no objective plaque index was recorded, which also constrained interpretation of the potential mechanisms underlying the model [33, 34]. Baseline caries was analyzed as a binary variable rather than as a continuous dmft score, which may have obscured a gradient in disease severity [15, 30].

## Conclusion

This cohort study of children aged 1–2 years in Beijing further elucidates the multifactorial etiology of early childhood caries (ECC) and highlights baseline caries status as a strong determinant of risk. We observed a modifying effect of baseline status on multiple cariogenic factors, which may help to optimize risk-stratified early intervention strategies and promote personalized oral health management from the earliest stages of life.

## Data Availability

The data presented in this study are available on request from the corresponding author.
